# All-Digital Time-Domain CMOS Smart Temperature Sensor with On-Chip Linearity Enhancement

**DOI:** 10.3390/s16020176

**Published:** 2016-01-30

**Authors:** Chun-Chi Chen, Chao-Lieh Chen, Yi Lin

**Affiliations:** Department of Electronic Engineering, National Kaohsiung First University of Science and Technology, Kaohsiung 81146, Taiwan; frederic@nkfust.edu.tw (C.-L.C.); u0252807@nkfust.edu.tw (Y.L.)

**Keywords:** CMOS, smart temperature sensor, time domain, linearity enhancement, field programmable gate array (FPGA)

## Abstract

This paper proposes the first all-digital on-chip linearity enhancement technique for improving the accuracy of the time-domain complementary metal-oxide semiconductor (CMOS) smart temperature sensor. To facilitate on-chip application and intellectual property reuse, an all-digital time-domain smart temperature sensor was implemented using 90 nm Field Programmable Gate Arrays (FPGAs). Although the inverter-based temperature sensor has a smaller circuit area and lower complexity, two-point calibration must be used to achieve an acceptable inaccuracy. With the help of a calibration circuit, the influence of process variations was reduced greatly for one-point calibration support, reducing the test costs and time. However, the sensor response still exhibited a large curvature, which substantially affected the accuracy of the sensor. Thus, an on-chip linearity-enhanced circuit is proposed to linearize the curve and achieve a new linearity-enhanced output. The sensor was implemented on eight different Xilinx FPGA using 118 slices per sensor in each FPGA to demonstrate the benefits of the linearization. Compared with the unlinearized version, the maximal inaccuracy of the linearized version decreased from 5 °C to 2.5 °C after one-point calibration in a range of −20 °C to 100 °C. The sensor consumed 95 μW using 1 kSa/s. The proposed linearity enhancement technique significantly improves temperature sensing accuracy, avoiding costly curvature compensation while it is fully synthesizable for future Very Large Scale Integration (VLSI) system.

## 1. Introduction

Thermal management systems (TMSs) are frequently used to manage the temperature of devices and equipment to prevent thermal damage [1]. The lifespans of components decrease when their temperature surpasses the safe temperature range, eventually causing damage and system breakdown. Temperature sensors are used to monitor temperature and send temperature information to TMSs. To ensure cost effectiveness and facilitate direct temperature monitoring, sensors can be mounted close to crucial heat sources and implemented in a complementary metal-oxide semiconductor (CMOS) process and, thus, be easily integrated with other Very Large Scale Integration (VLSI) circuits. Because of the fast growth in the circuit density and clock frequency, current VLSI systems, such as microprocessors [1,2,3], suffer from severe challenge in the temperature monitoring and control. Thus, the market demand for integrated temperature sensors for many industrial and home applications has increased substantially. Scientists integrate sensors into numerous systems since sensors become essential and tiny while consuming low energy. For precise monitoring, integrated temperature sensors require careful calibration to accurately read the temperature before use in TMSs because of process variations and silicon aging in integrated circuits (ICs).

To produce digital codes for communicating with VLSI systems, analog-to-digital converters (ADCs) have been integrated into temperature sensors to create “smart temperature sensors” [4,5,6,7,8]. Temperature sensors based on bipolar junction transistors are utilized to translate the temperature to a voltage signal proportional to the absolute temperature (PTAT), and then an ADC is used for digital temperature readings. Sophisticated circuits using additional on-chip calibration techniques are frequently used to ensure accuracy and full compatibility with the standard CMOS process [2,3,4,5,6,7,8]. Excellent performance, specifically, an extremely high accuracy of ±0.1 °C (3σ) and a low power dissipation of tens of microwatts, has been achieved. Offset cancellation techniques, dynamic element matching [5,7], and linearity enhancement techniques [6,8] are usually used; however, the circuit complexity and area increases substantially to be unattractive to the low-cost TMS.

**Figure 1:**
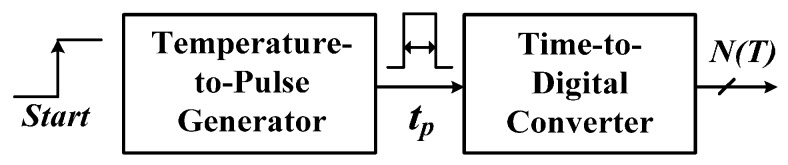
Architecture of first time-domain smart temperature sensor.

Time-domain CMOS smart temperature sensors [9,10,11,12,13,14,15,16,17,18,19] have been developed to reduce the circuit area and complexity substantially compared with that of voltage-domain smart temperature sensors. The first CMOS time-domain sensor consisted of a temperature-to-pulse generator with inverter-based delay lines and a time-to-digital converter (TDC) [9], as shown in Figure 1. CMOS inverters (NOT gates) have been used to sense temperature innovatively [9,10,11,12,13,14,15,16,17,18,19]. The sensor had an error of −0.7–0.9 °C after two-point calibration in a range of 0 °C–100 °C. With such simple structure, the small chip size and the satisfactory accuracy were achieved. Inverter-based oscillators of which the PTAT period can be transformed into temperature information are frequently implemented in time-domain temperature sensors. A temperature sensor implemented using two current-starved oscillators reached an inaccuracy of −1.6–3.0 °C in a range of 0–100 °C [10]. Another temperature sensor with a differential PTAT delay generator using a linear MOS operation had an error of −0.8–1.0 °C in a range of 0 °C to 100 °C [11].

**Figure 2:**
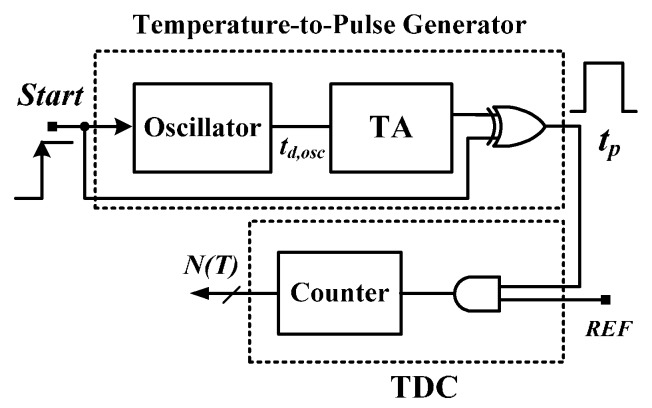
Architecture of oscillator-based smart temperature sensor.

To eliminate the burden associated with full custom designs, an all-digital smart temperature sensor implemented using Field Programmable Gate Arrays (FPGAs) was proposed for rapid prototyping [12]. As shown in Figure 2, an oscillator composed of inverters is first used to generate the oscillation period (*t_d,osc_*) PTAT. To achieve satisfactory sensor resolution, a time amplifier (TA) comprising a circulation counter is then used to amplify the *t_d,osc_*. An adequately wide pulse (*t_p_*) is obtained using a simple XOR gate. A TDC is composed of the reference period width (*t_REF_*), the AND gate, and the counter to convert the *t_p_* into the digital code. The smart sensor was realized with 140 logic elements (LEs) in FPGA chips and had an inaccuracy of −1.5–0.8 °C after two-point calibration in a range of 0–75 °C, showing that time-domain temperature sensors are suitable for cell-based or all-digital CMOS designs. However, two-point calibration, which can compensate for both gain and offset errors, needs to be used to reach an acceptable inaccuracy at the cost of test costs and time [9,10,11,12]. One-point calibration halves the test cost and time of two-point calibration for high-volume production; thus, it is more attractive in the market.

**Figure 3:**
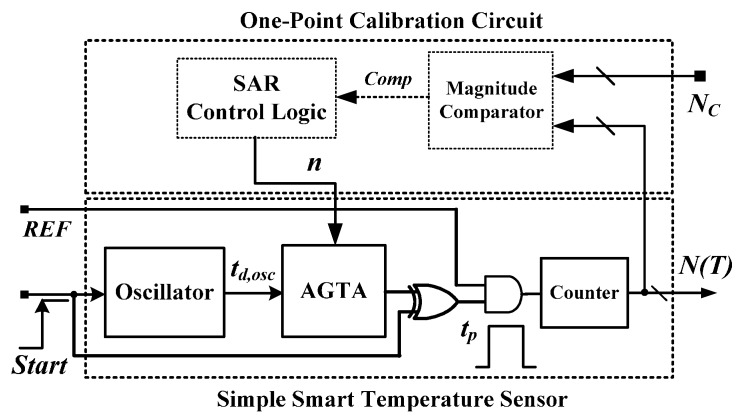
Architecture of smart temperature sensor with one-point calibration support.

Another FPGA version with one-point calibration support was invented and its architecture is presented in Figure 3 [13]. The structure of the simple smart temperature sensor is the same as that of [12]. Differently, an off-chip calibration circuit and an adjustable-gain TA (AGTA) were used to reduce the influence of process variations. Thus, one-point calibration can be used to save the test cost. However, the sensor has poor linearity because of the curvature caused by the CMOS inverter [9,10,11,12,13,14,15,16,17,18,19]. To overcome the problem of curvature, costly off-chip second-order master curve fitting was used for curvature correction to achieve a satisfactory accuracy of −0.7–0.6 °C after one-point calibration in a range of 0–100 °C [13]. Excluding the SAR-based (successive approximation register) calibration circuit, only 48 LEs for the simple smart temperature sensor were used.

A cell-based temperature sensor based on inverters was invented for self-calibration to eliminate the influence of process variations [14]. When no second-order master curve fitting was applied, the temperature sensor exhibited a substantial inaccuracy of −5.1–3.4 °C after one-point calibration in a range of 0–60 °C. Without applying the curve fitting, a frequency-based temperature sensor achieved a large inaccuracy of −2.8–2.9 °C after one-point calibration in a range of −40 °C to 110 °C [15]. Later research by the same team, a CMOS temperature sensor based on a process-variations-compensated frequency-to-digital converter was proposed [16]. The accuracy was enhanced by linearizing the frequency to digital conversion. Under one-point calibration, the achieved accuracy is scaled ±1.5 °C when measuring from −40 °C to 110 °C.

To enhance on-chip linearity without adopting the off-chip second-order master curve fitting, two delay lines were used at the cost of two-point calibration for curvature compensation, yielding the currently optimal inaccuracy of −0.35–0.3 °C from 0 to 90 °C [17]. To ensure acceptable inaccuracy in wider temperature range operations, another curvature compensation technique that involves using two oscillators was proposed. The sensor adopted two-point calibration to achieve a maximum inaccuracy of 1.4 °C for −40–120 °C extensive range [18].

Either costly off-chip second-order master curve fitting [13] or a time-consuming analog linearization technique [17,18] must be adopted to enhance linearity and achieve acceptable inaccuracy because of the curvature of CMOS inverters. Otherwise, the inaccuracy is substantial [14,15] or the operating temperature range is limited [12,14]. This study proposes the first all-digital on-chip linearity enhancement technique. Furthermore, applying the calibration technique described in [13] enables the fully digital smart temperature sensor to achieve a satisfactory accuracy after one-point calibration for a wider temperature range. The rest of this paper is organized as follows: Section 2 provides details on the proposed sensor, including the calibration circuit and the linearity enhancement technique; Section 3 presents the Experimental results; and finally, Section 4 concludes the paper.

## 2. Circuit Description

**Figure 4:**
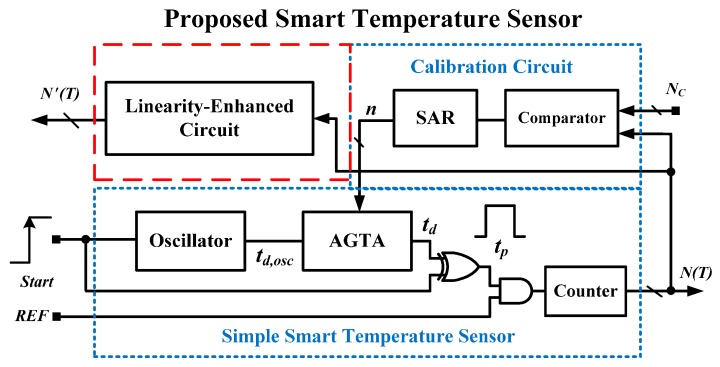
Architecture of the proposed all-digital smart temperature sensor.

The architecture of the proposed all-digital smart temperature sensor is presented in Figure 4. It comprises a simple smart temperature sensor, a calibration circuit for one-point calibration support, and a proposed linearity-enhanced circuit. The main feature that differentiates the proposed sensor from the time-domain sensors proposed in previous studies is the first all-digital on-chip linearity enhancement technique proposed in this paper. In contrast with the structure shown in Figure 3, the calibration circuit is implemented on-chip, and the linearity-enhanced circuit is connected with the simple version to linearize the digital value *N*(*T*) and derive a new digital value *N'*(*T*). Process variation calibration and linearity enhancement enable improving accuracy and reducing the test costs of high-volume production.

The proposed technique was introduced simply in [19]. The linearity-enhanced circuit and the calibration circuit were implemented on one FPGA board to off-chip calibrate and linearize simple smart temperature sensors fabricated in a 0.35-μm CMOS process. In this paper, the proposed sensor with calibration circuit and linearity-enhanced circuit was realized using the FPGA boards; a detailed description is provided.

### 2.1. Inverter-Based Smart Temperature Sensor and One-Point Calibration Technique

As mentioned previously, a CMOS NOT gate composed of a P-channel MOS and a N-channel MOS transistor can be used for temperature sensing to produce the PTAT delay. This can be expressed as [20]

(1) $$t_{NOT}(T)=\frac{2LC_{L}}{WC_{OX}V_{DD}}\times \frac{1}{\mu (T)}\times \frac{\ln(3-4V_{th}(T)/V_{DD})}{1-V_{th}(T)/V_{DD}}$$

where *T*, *W/L*, and *C_L_* are the operation temperature, aspect ratio of transistors, and loading capacitance of the NOT gates, respectively. The temperature relationship of mobility (*μ*) and threshold voltage (*V_th_*) are expressed as [21]

(2) $$\mu (T)=\mu_{0}\times {\left(\frac{T}{T_{0}}\right)}^{km},km=-1.2\sim -2.0$$

(3) $$V_{th}(T)=V_{th}(T_{0})+\alpha (T-T_{0}),\alpha =-0.5\sim -3mV/{}^{\circ}C$$

where *T_0_* is the reference temperature. Although *μ* and *V_th_* are both affected by the temperature, the thermal influence of *t_NOT_*(*T*) is dominated by *μ* [9,10,11,12,13,14,15,16,17,18,19]. Therefore, the thermal relationship of *V_th_* *(T)* can be ignored for simplification, and *t_NOT_*(*T*) can be further expressed as

(4) $$t_{NOT}(T)=\frac{2LC_{L}{T_{0}}^{km}}{\mu_{W}^{0}C_{OX}V_{DD}}\times \frac{\ln(3-4V_{th}/V_{DD})}{1-V_{th}/V_{DD}}\times \frac{1}{T^{km}}=\gamma \times T^{-km}$$

where *γ* is a process-dependent factor with nearly temperature independence. In Equation (4), the PTAT delay is dependent on the process variations. Furthermore, the oscillator constructed by the NOT gates resembles the NOT gate and can be used to generate an oscillation period PTAT, which is expressed as

(5) $$t_{d,osc}(T)=2\times k\times t_{NOT}(T)=2\times k\times \gamma \times T^{-km}$$

where *k* is the number of stages in the oscillator. The period is usually too short to attain a desired temperature resolution, unless *k* is set to an extremely high value. Thus, a TA with a gain of *n* was designed to amplify *t_d,osc_* and obtain the *t_p_* PTAT. Finally, the reference clock (*t_REF_*) was used to count *tp* to output the sensor output *N(T)*, which can be formulated as

(6) $$N(T)=\frac{t_{p}(T)}{t_{REF}}=\frac{n\times t_{d,osc}(T)}{t_{REF}}=\frac{n}{t_{REF}}\times 2\times k\times \gamma \times T^{-km}=\beta \times T^{-km}$$

where *β* is a factor with process dependence and temperature insensitivity because *n*, *k*, and *t_REF_* are ideally temperature insensitive. The characteristic of *N*(*T*) unavoidably resembles that of the NOT gate. In Equation (6), if *km* is −1 (the ideal value), then *N*(*T*) is perfectly linear. However, the value of *km* rangers from −1.2 to −2.0 for a 0.35-μm CMOS process [21], indicating that the transfer curve of *N*(*T*) has curvature.

**Figure 5:**
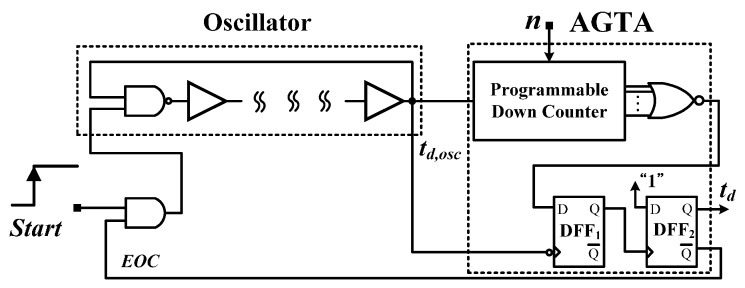
Circuits of the oscillator and the AGTA.

As shown in Figure 5 [13], the oscillator consists of a NAND gate and 24 NOT gates to produce the *t_d,osc_* PTAT. The AGTA consists of a programmable down counter and D type Flip Flops (DFFs) to amplify the *t_d,osc_*. The DFF_1_ was used for deglitching. Through the simple AND gate, an end-of-conversion (EOC) signal was activated to stop the oscillator for power saving.

Compared with the simple version, two parts were modified to support one-point calibration; an AGTA rather than the fixed-gain TA was used, and the calibration circuit composed of a magnitude comparator and SAR control logic was added. As described, the calibration technique used in this study is similar to that used in [13], in which the theory and calibration procedure were introduced. The temperature of the *i*th sensor was set to the calibration temperature (*T_C_*), and the corresponding *N(T)* of the sensor was varied by adjusting the time gain (*n*) of the AGTA to approximate the preset calibration value (*N_C_*). A magnitude comparator was used to detect the difference between *N(T)* and *N_C_*. The result, *Comp*, was sent to the SAR logic to set *n* to the proper value (*n_i_*) at *T_C_*. The variation in the oscillation period (*t_d,osc,i_*) of the *i*th sensor at *T_C_* was compensated properly to obtain the identical *N_C_* for all sensors. After calibration, all calibrated sensors have similar digital codes at the corresponding temperature because sensor resolution is stable (gain calibration) and all sensors have the same values at *T_C_* (offset calibration).

### 2.2. Proposed All-Digital On-Chip Linearity Enhancement Technique

Form Equation (6), because *km* is nearly constant for a given process technology [13,18], almost identical curvature is expectable among the error curves of the all calibrated sensors. According to the character of the similar curvature, individual linearization for each sensor is not required. Thus, the transfer curve of only one sensor is required to design the linearity-enhanced circuit, which linearizes the output curves of all sensors to attain the new curves with linearity improvement.

**Figure 6:**
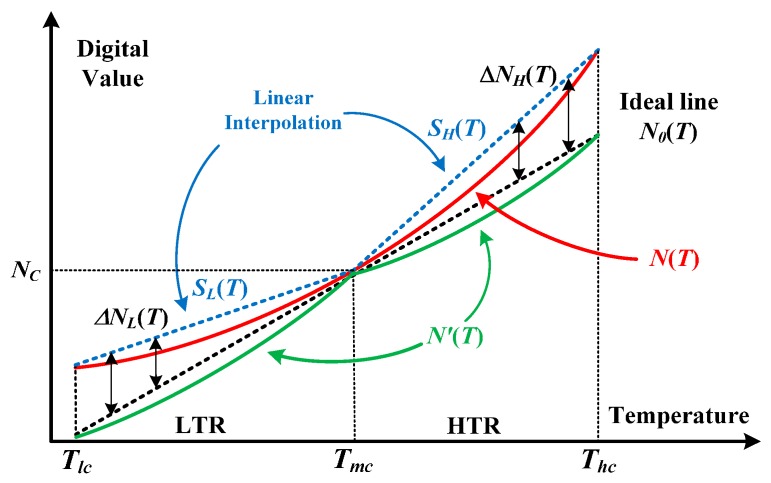
Concept of the linearity enhancement technique.

Figure 6 illustrates the concept of the proposed linearity enhancement technique. One transfer curve of the calibrated sensor is denoted as the *N*(*T*) curve. In addition to the *N_C_* at the medium calibration temperature *T_mc_* (*i.e.*, *T_C_* at the one-point calibration step), the two digital values *N*(*T_lc_*) and *N*(*T_hc_*) at the highest and lowest temperatures (*T_hc_* and *T_lc_*) were measured. Subsequently, according to the three known values *N_C_* = *N*(*T_mc_*), *N*(*T_hc_*) and *N*(*T_lc_*), we perform linear interpolation, *S_H_*(*T*) and *S_L_*(*T*), for estimating the error between the *N*(*T*) and the ideal line *N*_0_(*T*). Therefore, the error *N*(*T*)–*N_0_*(*T*) in the high-temperature region (HTR) with temperature *T* > *T_mc_* is approximated with

(7) $$\Delta N_{H}(T)=S_{H}(T)-N_{0}(T)$$

Then, the new output curve is calibrated as

(8) $$N^{\prime }(T)=N(T)-\Delta N_{H}(T)=N(T)-\left(S_{H}(T)-N_{0}(T)\right)$$

Similarly, for the low-temperature region (LTR), the new output curve is calibrated as

(9) $$N^{\prime }(T)=N(T)-\Delta N_{L}(T)=N(T)-\left(S_{L}(T)-N_{0}(T)\right)$$

The effect of the linearization regarding the curvature is obvious by observing the following facts. The maximum errors of *N*(*T*) in the HTR and LTR are respectively

(10) $$N(T_{hc})-N_{0}(T_{hc})=S_{H}(T_{hc})-N_{0}(T_{hc})$$

(11) $$N(T_{lc})-N_{0}(T_{lc})=S_{H}(T_{lc})-N_{0}(T_{lc})$$

while the ones of *N'*(*T*) in the HTR and LTR are smaller since derived from Equations (11) and (12) we have

(12) $$\left|N^{\prime }(T)-N_{0}(T)\right|=\left|N(T)-S_{H}(T)\right|=S_{H}(T)-N(T)<S_{H}(T)-N_{0}(T)<S_{H}(T_{hc})-N_{0}(T_{hc})$$

for *T_mc_* < *T* < *T_hc_* and

(13) $$\left|N^{\prime }(T)-N_{0}(T)\right|=\left|N(T)-S_{L}(T)\right|=S_{L}(T)-N(T)<S_{L}(T)-N_{0}(T)<S_{L}(T_{lc})-N_{0}(T_{lc})$$

for *T_lc_* < *T* < *T_mc_*.

The linear error approximation exhibits a curvature reduction with a noticeable linearity improvement in the new transfer curve.

**Figure 7:**
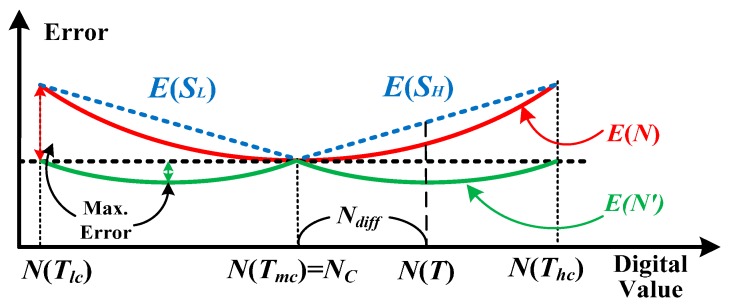
Error curves *versus* temperature with and without and the linearization.

Presented in Figure 7 are the corresponding error curves $E(N)=N(T)-N_{0}(T)$ and $E(N)=N_{0}(T)-N^{\prime }(T)$ along with digital value at different temperature. After the procedure of one-point calibration, the error at *T_mc_* is zero, and the maximal error in *E*(*N*) is greater than the one in *E*(*N′*). The error lines of linear approximation *E*(*S_H_*) for HTR and *E*(*S_L_*) for LTR can be respectively formulated as

(14) $$E(S_{H})=\frac{E(T_{hc})-E(T_{mc})}{N(T_{hc})-N(T_{mc})}\left(N(T)-N(T_{mc})\right)=\frac{E(T_{hc})}{N(T_{hc})-N_{C}}(N(T)-N_{C})$$

(15) $$E(S_{L})=\frac{E(T_{lc})-E(T_{mc})}{N(T_{mc})-N(T_{lc})}\left(N(T_{mc})-N(T)\right)=\frac{E(T_{lc})}{N_{C}-N(T_{lc})}(N_{C}-N(T))$$

where *E(T_hc_),* *E(T_lc_),* and *E(T_mc_*) are the errors of digital value at *T_hc,_* *T_lc,_* and *T_mc_,* respectively, and *E(T_mc_*) = 0 as mentioned. Theoretically, the linearized errors at *T_lc_*, *T_mc_*, and *T_hc_* should be zero.

**Figure 8:**
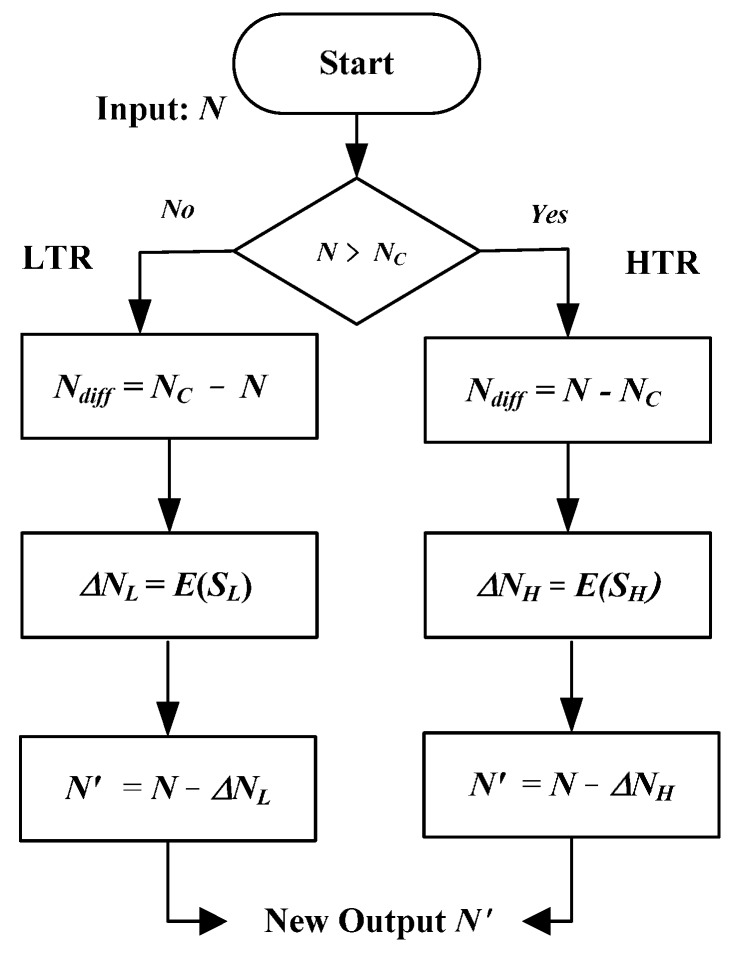
Flowchart for the linearization procedure.

As mentioned, the transfer curves of the calibrated sensors before linearization are almost the same, the proposed linearity-enhanced circuit is designed according to one sensor’s transfer curve, and it is applicable for linearization of other sensors to produce new curves with reduced curvature. According to Equations (14) and (15), the flowchart for the linearization procedure is presented in Figure 8. The first step is to determine the falling region of the algorithm input *N* read out from the calibrated sensor. When *N* is large than *N_C_*, it falls into HTR for the linearization. Contrarily, *N* falls into the LTR because it is smaller than *N_C_*. Then, the difference *N_diff_* between *N* and *N_C_* is calculated. By linear approximations of the digital value errors in Equations (14) and (15), the correction either $\Delta N_{H}=E(S_{H})$ or $\Delta N_{L}=E(S_{L})$ is determined in the third step. Finally, the linearized value $N^{\prime }=N-\Delta N_{H}$ or $N^{\prime }=N-\Delta N_{L}$ is attained.

**Figure 9:**
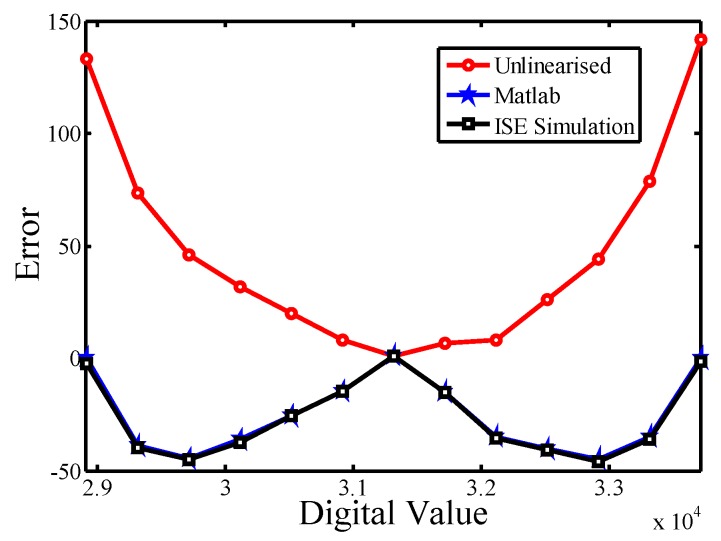
Inaccuracy of the unlinearized sensor and estimated inaccuracies of theoretical model using Matlab and ISE simulation for the proposed linearization.

To demonstrate the performance of the linearization, theoretical modeling using pure software (MATLAB) and simulation using the FPGA EDA (Electronic Design Automation) tool (Xilinx (Integrated Software Environment) ISE) were performed. The estimated inaccuracies are shown in Figure 9 for comparison. Compared with the unlinearized version, the error is improved considerably. The linearized results from theoretical modeling using MALTAB and ISE simulation were similar and resembled the expected effect *(i.e.*, *E*(*N*′)). This successfully validates the function of the proposed all-digital linearity enhancement technique. Linearity enhancement enables improving the accuracy of the sensor at low-cost condition, and neither costly off-chip second-order master curve fitting nor time-consuming analog signal linearization are necessary.

**Figure 10:**
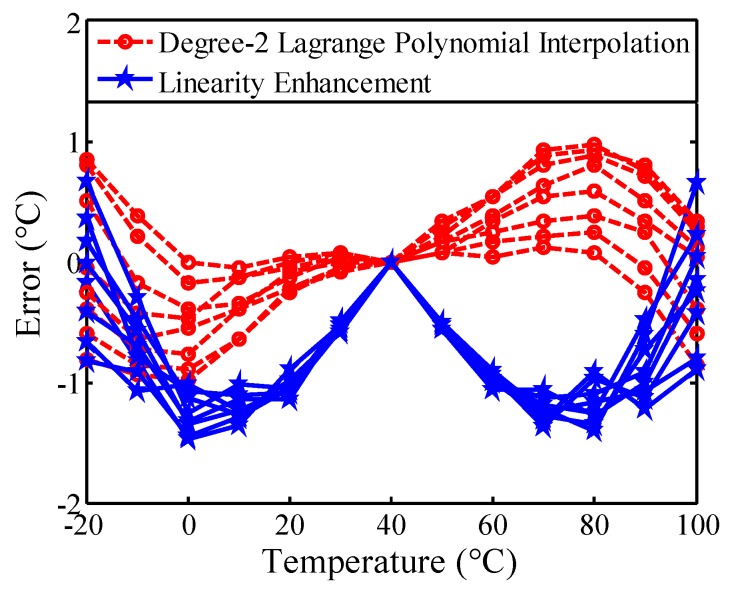
Estimated inaccuracies of the two techniques considering process variations of eight sensors.

The proposed linearity enhancement is easy to implement in a simple circuitry, which is more cost-effective compared to higher degree interpolation. The circuit area costs of the proposed linearity enhancement and degree-2 Lagrange polynomial interpolation technique [22] are 43 and 70 slices, respectively, while the respective accuracy ranges are −1.5–0.7 °C and −1–1 °C. By applying to eight sensors before linearization, the simulated results after improvement by the two techniques using ISE simulation tool are shown in Figure 10. The degree-2 method reaches a little bit better accuracy, but it noticeably requires much higher circuit area cost and design complexity.

In IC design, we usually utilize FPGA for ASIC (Application-Specific Integrated Circuit) fast prototyping. For pure digital design, the cell-based design flow is usually applied. Though the same linearity enhancement is also applicable to sensors designed by a full-custom flow, we usually do not compare area cost between cell-based ASICs and full-custom ones. For cell-based digital signal processing IC implementations, we usually express area cost in terms of number of arithmetic operations or in terms of basic components such as gates in ASIC or slices in FPGA. Traditional digital curve-fitting circuits require much more arithmetic operations especially when we were exploiting higher-degree polynomials for better accuracy. Due to a large amount of transistor counts, high degree curve fittings or interpolations are few implemented by full-custom design flow. In this paper, we recruit linear approximation which has successfully enhanced the accuracy using a small number of slices. It is possible to further improve the accuracy by using higher degree polynomial approximations. However, the number of arithmetic operations will also increase exponentially. The optimization of high order interpolation is another challenge.

## 3. Experimental Results

**Figure 11:**
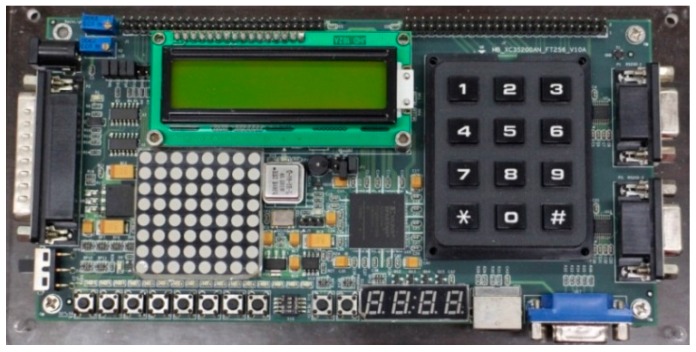
Photograph of the test FPGA board.

**Figure 12:**
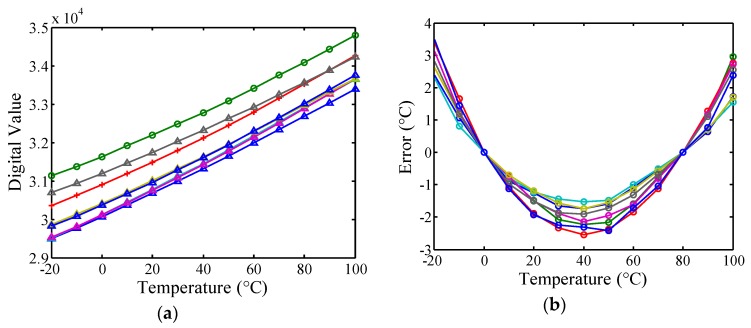
(**a**) Measured transfer curves of the simple smart temperature sensor for eight FPGA chips; (**b**) Inaccuracies of the simple smart temperature sensor after two-point calibration.

To evaluate the performance, a Xilinx XC3S200AN FPGA board with a low reference clock frequency of 40 MHz was used to realize the sensor, as shown in Figure 11. A total of eight FPGA chips for the simple smart temperature sensor with 16 output bits (*i.e.*, simple version without uncalibration and unlinearizion) were implemented to examine the influence of process variations, and the logic utilization for a single sensor is 50 slices. The measurements were performed in 10 °C steps in a range of −20 °C to 100 °C by using a programmable temperature and humidity chamber (MHG-120AF). The operating range was limited to 100 °C because the plastic devices on the board are easily damaged when the temperature is above 100 °C. The measured transfer curves of the simple version are presented in Figure 12a. The gain and offset errors caused by process variations are evident. Thus, two-point calibration was adopted to derive an acceptable inaccuracy. The temperature resolution ranges from 0.028 to 0.033 °C, and the large inaccuracy of −2.5–3.5 °C from −20 °C to 100 °C is exhibited, as presented in Figure 12b. As mentioned previously, to reduce test costs and time, a calibration technique for one-point calibration support was adopted in this study.

**Figure 13:**
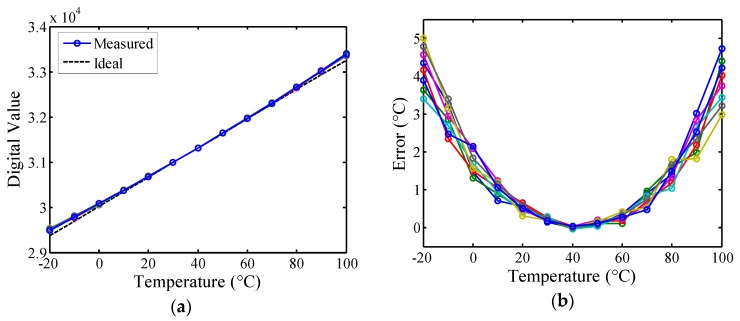
(**a**) Measured transfer curves of the *N(T)* for eight FPGA chips after calibration and ideal curve for comparison; (**b**) Inaccuracies of the calibrated (unlinearized) sensor after one-point calibration.

To verify the calibration performance, the logic utilization of 75 slices for a calibrated sensor (*i.e.*, unlinearized version) is implemented on the same FPGA board. Excluding the simple version, 25 slices were used for the calibration circuit. The measured transfer curves of the *N*(*T*) for the eight FPGA chips and the ideal curve are shown in Figure 13a for comparison. The *N*(*T*) of these chips nearly coincided at *T_C_* and changed linearly with the stable resolution as the temperature varied. Compared with the simple version, the gain and offset errors of the calibrated version decreased substantially, showing that the influence of process variations was effectively reduced to support one-point calibration. After one-point calibration, the corresponding inaccuracies are 5 °C in a range of −20 °C to 100 °C, as shown in Figure 13b. The curvature is apparent in the error curves, which conforms to Equation (6). Because of the curvature, greater inaccuracy is expected for wider temperature ranges [13,18]. If the curvature can be reduced or linearized, then the accuracy improves correspondingly. Therefore, this paper proposes the first all-digital on-chip linearity enhancement technique for reducing the curvature and improving accuracy.

**Figure 14:**
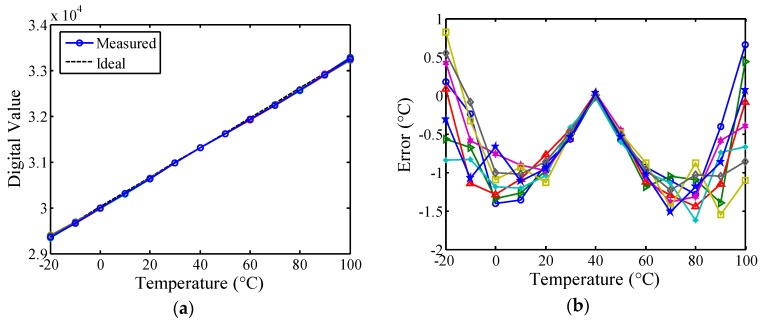
(**a**) Measured transfer curves of the *N'(T)* for eight FPGA chips after linearization and ideal curve for comparison; (**b**) Inaccuracies of the linearized sensor for eight FPGA chips.

To determine the feasibility of the proposed technique, the proposed sensor was realized using 118 slices in eight FPGA chips without employing any full custom devices, increasing flexibility, portability, and simplicity. The calibration circuit, which is useless after calibrating a sensor, can be implemented off-chip to reduce the circuit cost. Only 43 slices were used for the linearity-enhanced circuit. After linearization, the measurement results and the ideal curve are presented in Figure 14a for comparison. Compared with the unlinearized sensor, lower inaccuracies of −1.6–0.9 °C from −20 to 100 °C were achieved, as presented in Figure 14b. The measured error curves were similar to the expected curves, *E*(*N*′), and the estimated results from theoretical modeling using MALTAB and ISE simulation. The measurement results demonstrated that the proposed method, which features a fully digital CMOS design, functions favorably. The sensor had a resolution of 0.03 °C, and its power dissipation was measured as 95 μW at 1 kHz sampling rate and 3.3 V supply. A comparison among the measured performances of the simple version, the unlinearized version, and the proposed work is summarized in Table 1.

**Table 1:** Measured performances of the three versions for easy comparison.

Sensor	Resolution (°C)	Error (°C)	Calibration	Area (Slices)	Range (°C)	Technology (µm)
Simple	0.028 ~ 0.033	−2.5 ~ 3.5	Two-point	50	−20 ~ 100	0.09
Calibrated (Unlinearized)	0.03	0 ~ 5	One-point	75	−20 ~ 100	0.09
Linearized	0.03	−1.6 ~ 0.9	One-point	118	−20 ~ 100	0.09

Basically, we exploit signal processing technique to ease the design of accurate temperature sensors. Traditionally, to design an accurate temperature sensor, some sophisticated techniques either based on analog or digital circuit design principles are required. Then, the trimming would also become challenging. If we further consider the yield of manufacturing, the cost would be more uncertain. In this paper, the linearity-enhanced circuit is part of the sensor producing the linearized output *N*′(*T*), and it occupies a small area. We successfully avoid costly curvature compensation or other complex accuracy improvement techniques such that the sensor is fully synthesizable for future VLSI integration. According to the results of simulation and experiment, we can see the accuracy of the temperature sensor is much improved by the linearity-enhanced circuit. Moreover, the on-chip enhancement enables online field calibration which was difficult for traditional sensors since deployed. If we were applying off-chip enhancement, we have to additionally record each sensor’s data for individual calibration. On the other hand, since FPGAs are usually used for fast prototyping of ASIC designs, the linearity-enhanced circuit is applicable not only to sensors realized by FPGAs but also to those realized by ASICs or custom ICs.

For the accuracy comparison between the sensor in this FPGA and the one in [19], the primary difference is the process technology. Therefore, the initial inaccuracy caused by the process technology is also different. In this paper, we improve the accuracy from 0–5 °C to −1.6–0.9 °C, while, in [19], we improved the 0.35-μm full-custom sensor from 0–3 °C to −0.85–0.65 °C. Both of the linearizations have achieved 50% enhancement. Thus, we prove that the on-chip linearity enhancement works well even we are calibrating an ASIC prototype, an FPGA manufactured with 90-nm process. Generally, calibrating a sensor of high-end process technology is much more difficult than calibrating one of traditional process technology.

## 4. Conclusions

An all-digital CMOS smart temperature sensor featured with on-chip calibration and linearity enhancement has been proposed. After calibration, the sensor can support one-point calibration for test-cost and time reduction. To enhance accuracy, the all-digital linearity enhancement technique is proposed. Only one sensor must be measured for designing the linearity-enhanced circuit. When linearization was applied, the measurement results showed that the maximal inaccuracy of 5 °C was improved to 2.5 °C from −20 to 100 °C, and a twofold improvement in accuracy was achieved. The proposed sensor was developed without adopting a time-consuming full custom design for performance estimation or function certification. It occupied 118 slices in FPGA chip, achieved a robust resolution of 0.03 °C, and consumed 95 μW at 1 kHz. The measured performances of the related time-domain sensors is listed in Table 2 for comparison. With one-point calibration and fully digital CMOS design, the sensor exhibited the acceptable inaccuracy without requiring the off-chip second-order master curve fitting. Future studies will focus on a more effective all-digital on-chip linearity enhancement technique for time-domain smart temperature sensors.

**Table 2:** Performance comparisons among related works.

Sensor	Type	Resolution (°C)	Error (°C)	Calibration	Power Consumption (µW)	Area (mm^2^)	Range (°C)	Technology (µm)
[9]	Analog	0.12 ~ 0.16	−0.7~0.9	Two-point	9 @5 Hz	0.175	0~100	0.35
[10]	Analog	0.3	−1.6~3.0	Two-point	0.22 @100 Hz	0.05	0~100	0.18
[11]	Analog	0.3	−0.8~1	Two-point	0.4 @1k Hz	0.032	0~100	0.18
[12]	Digital	0.058	−1.5~0.8	Two-point	8.4 @2 Hz	140 LEs	0~75	0.22/0.18
[13]	Digital	0.133	−0.7~0.6 ^#1^	One-point	175 @1k Hz	48 Les ^#2^	0~100	0.22/0.18
[14]	Digital	0.139	−5.1~3.4	One-point	150 @10k Hz	0.01	0~60	0.065
[15]	Analog	0.043	−2.7~2.9	One-point	400 @366k Hz	0.0066	−40~110	0.065
[16]	Analog	0.18	±1.5	One-point	500 @ 465k Hz	0.008	0~110	0.065
[17]	Analog	0.088 ~ 0.093	−0.25~0.35	Two-point	36.7 @10 Hz	0.6	0~90	0.35
[18]	Analog	0.043 ~ 0.047	−0.2~1.2	Two-point	23 @10 Hz	0.07	−40~120	0.35
This work	Digital	0.03	−1.6~0.9	One-point	95 @1k Hz	118 Slices	−20~100	0.09

^#1^ with off-chip second order curve fitting; ^#2^ without one-point calibration circuit.
